# Child Night Blindness and Bitot's Spots Are Public Health Problems in Lay Armachiho District, Central Gondar Zone, Northwest Ethiopia, 2019: A Community-Based Cross-Sectional Study

**DOI:** 10.1155/2020/5095620

**Published:** 2020-11-15

**Authors:** Ajebew Bantihun, Kedir Abdela Gonete, Azeb Atnafu Getie, Asmamaw Atnafu

**Affiliations:** ^1^Amhara Regional State, Central Gondar Zone, Lay Armachiho District, Tekeldingay Health Office, Ethiopia; ^2^Department of Human Nutrition, Institute of Public Health, College of Medicine and Health Sciences, University of Gondar, Gondar, Ethiopia; ^3^Department of Health Systems and Policy, Institute of Public Health, College of Medicine and Health Sciences, University of Gondar, Gondar, Ethiopia

## Abstract

**Background:**

Night blindness (XN) is a condition in which a person cannot see in dim light and is the earliest clinical manifestation of vitamin A deficiency. Globally, vitamin A deficiency is a public health problem in 122 countries, of which 45 countries have moderate to severe child night blindness. Therefore, this study is aimed at assessing the prevalence and associated factors of night blindness and Bitot's spot among children aged 24-59 months.

**Methods:**

A community-based cross-sectional study was employed from February to March 2019 among children aged 24-59 months in the Lay Armachiho District, Amhara region. A structured and pretested questionnaire was used for data collection. Descriptive summary statistics were used to describe the study population. Bivariate and multivariable logistic regression models were used to identify associated factors.

**Results:**

Out of 1007 children, 1.9% and 2.2% had night blindness and Bitot's spot, respectively. Illiterate mothers (AOR = 2.94; 95%CI = (1.12, 6.72)), age of 48 to 59 months (AOR = 9.81; 95%CI = (1.24, 77.36)), ≥4 family sizes (AOR = 4.52; 95%CI = (1.02, 19.90)), had diarrhea (AOR = 5.00; 95%CI = (1.73, 14.54)), and had a respiratory tract infection (AOR = 3.14; 95%CI = (1.02, 9.70)) were significantly associated with night blindness. Age of 48-59 months (AOR = 4.23; 95%CI = (1.13, 14.86)) and mothers who did not wash their hands after using the toilet (AOR = 3.02; 95%CI = (1.01, 9.13)) were predictor variables for Bitot's spots.

**Conclusion:**

The prevalence of night blindness and Bitot's spots was high. Child's age, mother's educational status, family size, diarrhea in the last 2 weeks, and respiratory tract infection in the last 2 weeks were predictive variables for night blindness. Besides, handwashing practice after using the toilet and child's age were significantly associated with Bitot's spot among children. Therefore, both night blindness and Bitot's spots are a public health problem and call for the attention of health professionals in primary health care facilities.

## 1. Introduction

Vitamin A is an essential nutrient needed in small amounts for the normal functioning of the visual system and maintenance of cell function for growth, epithelial integrity, red blood cell production, immunity, and reproduction. This essential nutrient cannot be synthesized by the body and must be provided through diet [1]. Night blindness (XN) is a condition in which a person cannot see in dim light, and it is the earliest clinical manifestation of vitamin A deficiency. Additionally, it is a sensitive and specific indicator of low serum retinol levels [1, 2].

Globally, vitamin A deficiency is a public health problem in 122 countries; among these, 45 countries have moderate to severe child night blindness, and these include Iraq, Sri Lanka, India, and Southeast Asia with 3.93%, 1.6%, 1.56%, and 1.2%, respectively [3]. In Africa, the prevalence of night blindness was 0.9% and Bitot's spot 1.4%. In a national-based study in Kenya, South Africa, Nigeria, Senegal, and Morocco, the prevalence of night blindness among children under five (1–5 years) was 2.0%, 1.6%, 1.0%, 0.36%, and 0.16%, respectively [4].

Based on a study conducted in 2005 in Ethiopia, the national prevalence of night blindness among children was 0.8% and the national prevalence of Bitot's spot among children was 1.7%, while the magnitude and distribution of child night blindness were highest in Harari (1.1%), followed by Beneshangul-Gumuz and Amhara (both 1.0%) [5].

According to the World Health Organization (WHO), the overall prevalence of night blindness (more than 1), Bitot's spots (>0.5%), corneal xerosis (>0.01%), and corneal scar (>0.05%) can be considered a public health problem for the community [6]. Serum retinol levels reflect liver vitamin A stores only when they are severely depleted (<0.07 *μ*mol/g liver) or extremely high (>1.05 *μ*mol/g liver) [7]. Between these extremes, serum retinol is homeostatically controlled and thus not always correlated with vitamin A intake or clinical signs of deficiency. Consequently, serum retinol is not useful for assessing the vitamin A status of individuals and may not respond to interventions. Rather, the distribution of serum retinol values in a population and the prevalence of individuals with serum retinol values below a given cutoff can provide important information on the vitamin A status of a population and may reflect the severity of vitamin A deficiency as a public health problem [6].

The primary cause of vitamin A deficiency is a lack of adequate intake of vitamin A and may be exacerbated by high rates of infection, especially diarrhea and measles. It is one of the most important causes of preventable childhood blindness and is a major contributor to morbidity and mortality from infections, especially in children and pregnant women, affecting the poorest segments of populations, particularly those in low- and middle-income countries [1]. Also, VAD is aggravated by lack of education, poor sanitation, absence of new legislation and poor enforcement of existing food laws, and lack of a monitoring and surveillance system [1].

Additionally, a diet with chronic insufficient vitamin A will affect the growth spurt of a child and aggravate illness that can affect appetite, absorption, transportation, and storage capacity. This greatly raises the risk of health consequences, like preventable child blindness, diarrhea, and respiratory tract infection, and increases mortality caused by vitamin A deficiency disorders (VADD) [8]. The most commonly available animal and plant source foods that contain significant quantities of the form of vitamin A that is functional in the body include cream; butter and nonskim milk; eggs; liver; fish; oils that are orange or red in color, such as red palm oil; noncitrus fruits that are yellow, orange, or red in color; root crops; squash and pumpkin; most green vegetables; and dark green leafy vegetables [9]. Even though Ethiopia has periodically delivered a high-potency vitamin A supplementation program for children aged 6-59 months old, vitamin A deficiency still remains a public health problem. Therefore, this study was conducted to determine the prevalence and associated factors of current night blindness and Bitot's spot among 24- to 59-month-old children.

## 2. Methods

### 2.1. Study Design and Setting

A community-based cross-sectional study design was employed from February to March 2019 in the Lay Armachiho District involving children aged between 23 and 59 months old.

Lay Armachiho is one of the 12 administrative districts in the central Gondar zone and is located at a distance of 22 kilometers from Gondar town. The weather conditions of the district are mostly “Weyna Dega” (weather conditions that are between cold and hot) and “kola” (a hotter weather condition). The common food products produced in the district are cereals and legumes including teff, barley, wheat, maize, sorghum, millet, chickpeas, peas, and beans. Banana, papaya, lemon, orange, carrot, onion, and potato are the other major categories of field crops in the district.

Currently, there are 6 health centers, 26 health posts, three junior private clinics, one medium private clinic, and two private drug stores providing health services for the community. In 2016/17, G.C. estimated population of the district was 143,436 of which 12,185 were children in the age range of 24–59 months old [10].

### 2.2. Study Population and Procedures

All children aged 24-59 months who were living in the district during the study period were included in the study. The sample size for the prevalence was calculated using a single population proportion formula with the assumption of a proportion of the previous study of 1.0% and 3.2% for night blindness and Bitot's spot among children in the Amhara region, respectively, using a 95% confidence interval, a margin of error of 2 for both, a design effect of 2, and 10% response rate. The second objective (determinant factors) was used as the variables; knowledge of a mother about the source of vitamin A (920) and having no functional latrine were calculated with a double population proportion formula [11]. The final minimum adequate sample size was 1012.

A multistage sampling technique was employed to select eligible participants for the study. From among the total 25 kebeles, five kebeles were selected using the lottery method. The numbers of households and eligible children in each selected kebeles were obtained from the district health office. Eventually, 1012 subjects were selected by a systematic random sampling technique from the households with a fixed number interval (*k*). If there was no child in the selected household, the next household with an eligible child was considered, and if there were more than one child with ages of 24-59 months in the household, one was selected randomly by the lottery method.

### 2.3. Operational Definitions

- Dietary diversity: among the seven food groups (dairy products, flesh foods, eggs, grains, legumes and nuts, roots and tubers, and vitamin A-rich fruits and vegetables), dietary diversity is said to be adequate if the child consumes at least one food item from each of four or more food groups within a 24 hour recall period [11].
- Night blindness: the study subjects were considered as having night blindness when they do not have a difficulty seeing during the day but have a difficulty seeing with decreased light or at night [12].
- Bitot's spot: the slightly elevated, white foamy lesion is usually seen as the temporal part of the bulbar conjunctiva near the limbus at the three o'clock or nine o'clock positions [13].
- A safe distance of water: is when the distance of the source of water from the house takes 30 minutes to complete a round trip [13].
- Health illness: refers to being ill over 15 days preceding the data collection [13].
- Literate: a person is literate who can, with understanding, both read and write a short, simple statement about his everyday life, whereas an illiterate is a person who is unable to read and write [14].

### 2.4. Data Collection Tool, Measurement, and Management

The questionnaire was first developed in English and then translated into Amharic and retranslated into English to check its consistency. Data was collected using a pretested and structured interviewer-administered questionnaire and physical examination of Bitot's spot. Based on these, data on sociodemographic and economic characteristics, diet-related factors, health-related factors, water and sanitation factors, and awareness of mothers on nutrition were collected.

The assessment of child night blindness (known by the local term “dafinit” in Amharic) was based on the reports from mothers regarding the condition of their children who do not have a difficulty seeing during daytime but have a difficulty seeing with decreased light or at night by using a standardized sequence of questions. Besides, a trained clinical nurse performed an eye examination on children who had a collection of the cheesy or foamy ocular lesion on conjunctiva and confirmed it as Bitot's spot [5].

According to the IMNCI guideline (MOH, 2005), children under 5, whose mothers were asked if the child had an experience of watery or loose stool 3 or more times per day and had an experience of cough accompanied by short and rapid breathing or difficulty of breathing as a result of chest-related problems, were considered to have diarrhea and acute respiratory infection (ARI), respectively [15].

The seven-day food-frequency questionnaire was administered to the respondents to determine how often vitamin A-rich foods were consumed preceding the survey. According to WHO indicators for assessing vitamin A deficiency [12], when vulnerable groups consume foods with high vitamin A content <3 times per week, there is a high risk of inadequate vitamin A status.

Data collection was performed by five clinical nurses and two supervisors who have BSc nurses also recruited for supervision. Both data collectors and supervisors were selected from the same district. The supervisors were assigned to assist the data collection process and carry out a reliability test on a small number of randomly selected subjects. Day-to-day supervision was conducted during the whole period of data collection. The overall activity was coordinated by the principal investigator. At the end of each day, the questionnaire was revised and checked for completeness and consistency of the data.

Data collectors and supervisors together with the ophthalmology nurse were given two days of training on how to interview and identify clinical features of vitamin A deficiency (night blindness and Bitot's spot). The pretest was conducted in another nearby district (Wogera) to assess questionnaires for its length, clarity, completeness, and consistency before the actual study period. The principal investigator and supervisors made day-to-day visits at the study site, supervised data collectors, and handled the questionnaires. At the end of each day, the collected data were checked for completeness and consistency by reviewing every participant's data. Appropriate feedback was given to data collectors before the next data collection day.

### 2.5. Data Processing and Analysis

Data were coded, cleaned, edited, and finally entered using epi.info version 7 and transported to SPSS version 20 for further analysis. Data cleaning was performed to check its accuracy, consistency, missed values, and variables. Descriptive statistics, such as frequency distribution, percentage, and mean and standard deviation, were used with tabular presentation for summarizing the findings. Both bivariate and multivariable logistic regression analyses were used to identify the factors associated with night blindness and Bitot's spot. Those variables that have *P* values < 0.2 in the bivariable analysis were fitted into the multivariable analysis. The results of *P* values < 0.05 in the multivariable logistic regression analysis were considered as statistically significant. The wealth index measurements were calculated using PCA (principal component analysis). During the analysis, Hosmer and Lemeshow's goodness-of-fit test was considered. Crude and adjusted odds ratios with 95% CI were calculated to see the strength of association of each independent variable with the dependent variable.

### 2.6. Ethical Considerations

Ethical clearance was obtained from the ethical review board of the University of Gondar. Permission and supporting letters were obtained from the district health office and administrative office. The purpose and importance of the study were explained to the participants. Data were collected after fully informed verbal consent and assent were obtained from the mothers, and confidentiality of the information was maintained by omitting their names and personal identification to retain privacy. Those subjects who refused to participate were respected. Children who were found to have night blindness and Bitot's spot were advised to visit the local health facility for intervention.

## 3. Result

### 3.1. Sociodemographic and Economic Characteristics

A total of 1007 children participated at a response rate of 99.5%. The mean ages of the children and their mothers were 42.47 ± 9.86 months and 30.05 ± 4.92 years, respectively. The birth interval of more than half (593 (58.9%)) of the children was more than three years. The majority of the respondents (989 (98.2%)) were married, and almost all (990 (98.3%)) were orthodox Christian followers. Nearly two-thirds of the children's mothers and fathers were illiterate (636 (63.2%) and 632 (62.8%), respectively). Subsistence farming (947 (94.0%)) was the main occupation of fathers, whereas housewife (985 (97.8%)) was the main occupation of mothers. Around two-thirds of the respondents (648 (64.3%)) have a family size of four and more, and about one-third (341 (33.9%)) of the respondents were poor in the wealth index (Table 1).

### 3.2. Water and Sanitation Characteristics of Respondents

Two-thirds of the respondents (633 (62.9%)) obtained their drinking water from unimproved water sources, and the majority (960 (95.3%)) of them did not treat their drinking water. Almost all (981 (97.4%)) of the study participants were spending less than 30 minutes to obtain their drinking water. More than half (588 (58.4%)) of the mothers did not wash their hands after using the toilet, and three-fourths (790 (78.5%)) of the respondents did not have latrines (Table 2).

### 3.3. Health Illness and Related Factors

Nearly one-tenth (82 (8.1%)) of the children had acute respiratory infection symptoms, and a few (72 (7.1%)) of the children experienced diarrhea two weeks before data collection. Among the children who had diarrhea for the last two weeks, 59 (5.9%) and 13 (1.2%) had watery and bloody diarrhea, respectively. About 23 (14.9%) of the children were treated immediately following illness, but more than one-third (58 (38.5%)) of the children were not treated during illness (Table 3).

### 3.4. Dietary Assessment

Among the overall participants, the majority (870 (86.4%)) of the children had inadequate dietary diversity scores. Nearly all (920 (91.4%)) children did not receive vitamin A supplementation in the last 6 months preceding the survey. Among the food groups, few (88 (8.7%), 62 (6.2%), and 45 (4.4%)) of the children consumed milk, green leafy vegetables, and fruits three or more times per week. Half (519 (51.5%)) of the respondents have consumed lipids (oil/fat). The majority (912 (90.5%)) of the study participants have never consumed meat in the last seven days preceding the data collection (Table 4).

### 3.5. Awareness of Vitamin A Source and Deficiency

Among 1007 study participants, 952 (95.5%) of them did not have information about the vitamin A nutrient. More than three-fourths (826 (82.0%)) of the participants did not believe vitamin A deficiency is prevented with foods (Table 5).

### 3.6. Factors Associated with Night Blindness

In the bivariate logistic regression analysis, it is illustrated that the age of the child, the sex of the child, mothers' educational status, fathers' educational status, family size, latrine availability, diarrhea, and respiratory tract infection in the last 2 weeks were significantly associated with night blindness at a *P* value < 0.2 level of significance, and these were entered into the multivariable logistic regression model. Consequently, in the multivariable analysis, children's age between 48 and 59 months, mothers' educational status, family size greater than or equal to four, diarrhea, and respiratory tract infection were found to be significantly associated with night blindness at a *P* value < 0.05. Children from illiterate mothers were 3 times more likely to have night blindness as compared to the children from literate mothers (AOR = 2.94, 95%CI = (1.12, 6.72)). The children from a familysize ≥ 4 were 4.5 times more likely to have night blindness as compared to children from a familysize < 4 (AOR = 4.52; 95%CI = (1.02, 19.90)). Children 48 to 59 months of age were 9.8 times more likely to have night blindness compared to children 36 to 48 months of age (AOR = 9.81; 95%CI = (1.24, 77.36)). Additionally, the children who did have diarrhea were 5 times more likely to have night blindness compared to children who did not have diarrhea (AOR = 5.00; 95%CI = (1.73, 14.54)). Furthermore, children who did have respiratory tract infection were 3 times more likely to have night blindness compared to children who did not have a respiratory tract infection (AOR = 3.29; 95%CI = (1.07, 9.70)) (Table 6).

### 3.7. Factors Associated with Bitot's Spot

Both bivariate and multivariate logistic regressions were applied to identify factors associated with Bitot's spot. Children's age, sex of the child, mother's educational status, family size, dietary diversity, and handwashing practices after using the toilet were predictor variables that were significantly associated with Bitot's spot at a *P* value < 0.2. In the final model, children's ages between 48 and 59 months and handwashing practices after using the toilet were found to be significantly associated with Bitot's spot at a *P* value < 0.05. In the multivariable analysis, children aged 48-59 months whose mothers did not practice handwashing after using the toilet were 3 times more likely to have Bitot's spot compared with children whose mothers did practice handwashing after using the toilet (AdjustedOddsRatio(AOR) = 3.02; 95%CI = 1.01, 9.13). Children between the ages of 48 and 59 months were 4 times more likely to have Bitot's spot compared to children between the ages of 36 to 47 months (AOR = 4.23; 95%CI = 1.13, 14.86) (Table 7).

### 3.8. Prevalence of Night Blindness and Bitot's Spot

**Figure 1:**
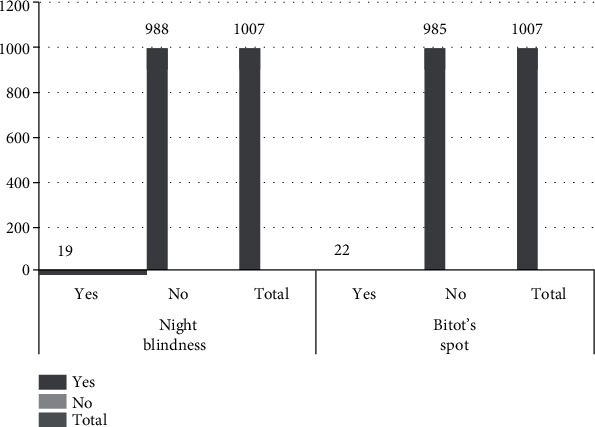
Diagrammatic presentation of the prevalence of night blindness and Bitot's spot.

The overall prevalence of night blindness and Bitot's spots were 19 (1.9% (95%CI = 1.1, 2.8%)) and 22 (2.2% (95%CI = 1.4, 3.1%)), respectively. Among the participants, 8 (0.79%) of them suffered from both night blindness and Bitot's spot (Table 8 and Figure 1).

## 4. Discussion

In preschool age children, night blindness is common in many developing countries. Globally, night blindness affects 5.2 million preschool age children, which correspond to 0.9% of the population at risk of VAD [3]. Almost half of the global preschool age children (2.55 million) affected with night blindness were found in Africa [3]. The prevalence of night blindness in school age children in Africa is 2.0%, which is four times higher than in Southeast Asia (0.5%) [3].

In this study, the overall prevalence of night blindness among children in the age range of 24-59 months was 1.9% (95%CI = (1.1, 2.8%)). This prevalence is almost two times higher than the WHO cutoff point for public health significance (1%) [3] and in line with the prevalence in Africa [3].

A possible explanation for this was a persistently low intake of vitamin A-rich foods and lack of knowledge about vitamin A-rich food sources. This community-based cross-sectional study finding was in line with the findings from the South Indian state Andhra Pradesh (1.3%) and Karnataka (2.0%) [16]. The similarities might be related to the similarity of study design and socioeconomic characteristics. Similarly, other studies were conducted in Ethiopia including Harari (1.1%) [5] and Tigray (1.2%) [17]. This might be due to the similar maternal feeding practice regarding vitamin A that was inadequate across the nation. But this finding is lower than the national survey report of Congo (4.3%) [3], Mali (5.2%) [3], and Sudan (4.2%) [3]. This huge discrepancy might be due to a large sample size compared to the national survey report of those countries [17] and due to having a low educational level of mothers compared to those of Congo, Mali, and Sudan. Uneducated mothers may not easily understand the consequences of a diet with poor vitamin A sources, especially a diet of animal origin [3]. According to previous studies, maternal education is one of the key determinant factors for night blindness [18]. However, the current finding was relatively higher than the findings from national studies conducted in Ethiopia: nationwide = 0.8%; Amhara = 1%; Benshangul gumuz = 1%, Afar = 0.9%, and Tigray = 0.9% [5]. This difference might have been due to the national studies done nationwide on a larger sample size. Besides, it was conducted on a culturally different population, which may have different child-feeding practices, while the current study was conducted on an almost culturally homogenous population with similar feeding practices.

In the current study of multivariable analysis, children aged 48 to 59 months were 9.8 times more likely to have night blindness compared to children aged 36 to 47 months. Similar findings were reported in Mali [3] and Sudan [3]. This is probably because this age category is the turning point for increased energy and micronutrient requirements compared to younger children, to support their rapid growth and development [19], and children in this age group are more noticeable by their mothers or family than younger children.

The current study also found that children from a family size of four and more were 4.5 times more likely to have night blindness as compared to children from a family size of less than four. This finding is consistent with studies done in Sudan, the Tigray region, and India [18]. This might be due to the reason that the big family size had reduced the children's share of energy-, protein-, iron-, and vitamin A-rich sources of foods and led to an increase in the chance of illness resulting in the reduced appetite of children for foods containing vitamin A, and in turn, causing night blindness as a manifestation of vitamin A deficiency [20].

In this study, the children from illiterate mothers were 3 times more likely to have night blindness as compared to the preschool children from literate mothers. It is consistent with a study in rural South India which reported that children from illiterate mothers were at higher risk for developing night blindness than children from literate mothers [21]. This could be because literate mothers had better information and knowledge about the source of vitamin A-rich foods, and they can feed their children with vitamin A-rich foods.

Moreover, the current study found that children who did have diarrhea in the last two weeks preceding the survey were 5 times more likely to have night blindness compared to children who did not have diarrhea. This finding is supported by that of another study conducted in India and Pakistan [22]. This could be related to diarrhea which can reduce vitamin A absorption and also cause loss of appetite. Also, it can increase metabolic demand that leads to protein energy malnutrition interfering with protein and with the storage, transport, and utilization of vitamin A [21].

Children who did have respiratory tract infection in the last two weeks preceding the survey were 3 times more likely to have night blindness compared to children who did not have respiratory tract infection. This finding is similar to that of a study conducted in India [21]. This might due to infection that can reduce appetite, and in addition to this, it can increase metabolic demand that leads to protein energy malnutrition interfering with protein and with the storage, transport, and utilization of vitamin A.

This community-based cross-sectional study identified that 2.2% (95%CI = (1.4, 3.1%)) of the children aged 24 to 59 months developed Bitot's spots which are around four times higher than the WHO cutoff point of considering VAD as a public health problem (0.5%) [23]. This finding was in line with the findings from India (Madhya Pradesh (1.4%) and Andhra Pradesh (2.71%) [23]); Mali (2.0%) [23]; and Ethiopia (national report (1.7%), Afar (2.1%), Oromia (1.5%), Addis Ababa (1.4%), and Alaje and Samre Weredas of Tigray (1.5%) [23]). In another way, the current finding was higher than the findings from Tigray (0.7%), Beneshangul-Gumuz (0.8%), Diredawa (1.1%), and Harari (1.2%) [21]. This might be because of the culturally and ecologically different populations and also because of a different study period that affects the vitamin A source food production [21]. However, this finding is lower than the national survey report which was found in the Amhara region (3.2%) [21]. This difference might be due to the large sample size, and our finding came from a study that was conducted in a homogenous population with similar dietary and health practices in the study area.

Children between the ages of 48 and 59 months and handwashing practices after using the toilet were found to be significantly associated with Bitot's spot at a *P* value < 0.05. Children between the ages of 48 and 59 months were 4 times more likely to have Bitot's spot compared to children aged 36 to 47 months. This might be due to longstanding vitamin A deficiency that is most prevalent in children aged 48 to 59 months [23]. This is also probably because children in this age category are more active and try to feed themselves and are exposed to the contamination that leads to diarrhea-induced vitamin A deficiency.

The children aged 24-59 months whose mothers did not practice handwashing after using the toilet were 3 times more likely to have Bitot's spot compared with children whose mothers did practice handwashing after using the toilet. This study was consistent with a study done in Bangladesh [24]. The reasons might be that poor hygiene and sanitation are well-defined underlying causes of malnutrition and not practicing handwashing after using the toilet and the lack of toilets to use influence households making them highly prone to infections due to contaminations that subsequently lead to disease-induced vitamin A deficiency [25]. Night blindness was assessed using an interview mechanism and this might have a subjective influence on the outcome.

## 5. Conclusion

This study reveals that the prevalence of night blindness and Bitot's spot among children aged 24 to 59 months were major public health problems. Children's age, mother's educational status, family size, diarrhea in the last 2 weeks, and respiratory tract infection in the last 2 weeks were predictors for night blindness and Bitot's spot among children aged 24 to 59 months. Handwashing practice after using the toilet and children aged 48 to 59 were predictors for Bitot's spot among children aged 24 to 59 months. Routine vitamin A supplementation had very low coverage in the study area and calls the attention of health professionals in primary health care facilities to strengthen vitamin A supplementation coverage and giving special emphasis to preschool children to tackle vitamin A deficiency.

## Figures and Tables

**Table 1:** Sociodemographic and economic characteristics among children aged 24-59 months in Lay Armachiho District, Central Gondar Zone, northwest Ethiopia, 2019.

Variables		Variables	Percent (%)
Age of children (in months)	24-35	295	29.3
	36-47	328	32.6
	48-59	384	38.1
Sex of children	Male	508	50.4
	Female	499	49.6
Birth interval	<3	414	41.1
	≥3	593	58.9
Age of mother (in years)	<20	34	3.4
	20-34	784	77.8
	35-49	189	18.8
Religion	Orthodox Christians	990	98.3
	Muslim	17	1.7
Ethnicity	Kimant	985	97.8
	Amhara	22	2.2
Mother's educational status	Illiterate	636	63.2
	Literate	371	36.8
Father's educational status	Illiterate	632	62.8
	Literate	375	32.2
Mother's occupation	Housewife	985	97.8
	Government employee	4	0.4
	Others	18	1.8
Father's occupation	Farmer	947	94.6
	Government employee	53	5.3
	Others	7	0.1
Marital status of the mother	Married	989	98.2
	Unmarried	2	0.2
	Separated	6	0.6
	Divorced	7	0.7
	Widowed	3	0.3
Number of children in the family	<4	359	35.7
	≥4	648	64.3
Wealth index	Poor	341	33.9
	Medium	327	32.4
	Rich	339	33.7

**Table 2:** Water source and hygienic characteristics among children aged 24-59 months in Lay Armachiho District, Central Gondar Zone, northwest Ethiopia, 2019.

Variables		Frequency	Percent (%)
Source of drinking water	Improved	374	37.1
	Unimproved	633	62.9
Time to obtain drinking water	<30	981	97.4
Round trip in a minute	≥30	26	2.6
Water treatment	Chlorine	3	0.3
	Boiling	44	4.4
	No treatment	960	95.3
Latrine availability	Yes	217	21.5
	No	790	78.5
	Yes	419	41.6
Handwashing practice after using the toilet	No	588	58.4

Note: unimproved drinking water including unprotected spring water, river water, and unprotected well water which is not suitable for drinking (EDHS 2016).

**Table 3:** Illness characteristics among children aged 24-59 months in Lay Armachiho District, Central Gondar Zone, northwest Ethiopia, 2019.

Variables		Frequency	Percent (%)
Diarrhea in the last two weeks	Yes	72	7.1
	No	935	92.9
Type of diarrhea	Bloody	13	1.2
	Watery	59	5.9
Respiratory tract infection in the last 2 weeks	Yes	82	8.1
	No	925	91.9
Both diarrhea and respiratory tract infection	Yes	154	15.3
	No	853	84.7
Children treated for both diarrhea and respiratory tract infection	Yes	96	62.3
	No	58	37.6
Time of treatment	Immediately	23	14.9
Following illness	After two days	44	28.6
	After three and more days	29	18.8
The reason why the child was not treated at the time of illness	Became well without treatment	40	68.9
	I did not have enough money	10	17.4
	I was busy	8	13.7

**Table 4:** Dietary characteristics among children aged 24-59 months in Lay Armachiho District, Central Gondar Zone, northwest Ethiopia, 2019.

Variables		Frequency	Percent (%)
Milk and milk products	One day	176	17.4
	Two day	113	11.2
	Three and more days	88	8.7
	None at all	630	62.6
Eating meat	One day per week	64	6.4
	Two days per week	30	3.0
	Three and more days per week	1	0.1
	None at all	912	90.5
Egg	One day per week	106	10.5
	Two days per week	85	8.4
	Three and more days week	38	3.7
	None at all	808	80.1
Green leafy vegetables and roots	One day per week	112	11.1
	Two days per week	89	8.8
	Three and more days per week	62	6.2
	None at all	744	73.9
Vitamin A-rich fruits	One day per week	134	13.3
	Two days per week	97	9.7
	Three and more days per week	45	4.4
Any oil/fats	None at all	731	72.6
	One day per week	25	2.5
	Two days per week	76	7.5
	Three and more days per week	519	51.5
	Daily	273	27.1
	None at all	114	11.3
Vitamin A supplementation in the last six months	Yes	87	8.6
	No	920	91.4
Dietary	Adequate	137	13.6
Diversity	Inadequate	870	86.4

**Table 5:** Awareness of vitamin A deficiency and consequence among children aged 24-59 months in Lay Armachiho District, Central Gondar Zone, northwest Ethiopia, 2019.

Variables		Frequency	Percent (%)
Vitamin A deficiency education in the last two weeks	Yes	96	9.6
	No	911	90.5
Having information on vitamin A nutrient	Yes	55	5.5
	No	952	95.5
Knowing the source of vitamin A	Animal only	25	1.49
	Plant only	17	1.29
	Both animal and plant	13	2.08
Cause of night blindness	Microorganisms	73	7.2
	Vitamin A deficiency	35	3.6
	Anemia	70	7.0
Night blindness is preventable	Yes	181	18.0
	No	826	82.0

**Table 6:** Results of bivariate and multivariable logistic regression of night blindness among children aged 24-59 months in Lay Armachiho District, Central Gondar Zone, northwest Ethiopia, 2019.

Variables		Night blindness		COR (95% CI)	AOR (95% CI)
		Yes (%)	No (%)		
Mother's educational status	Illiterate	16 (1.49%)	620 (61.66%)	3.17 (1.17, 7.69)	2.94 (1.12, 6.72)^∗^
	Literate	3 (0.39%)	368 (36.44%)	1.00	1.00
Father's educational status	Illiterate	15 (1.49%)	617 (61.27%)	2.25 (0.74, 6.85)	2.07 (0.67, 6.42)
	Literate	4 (0.39%)	371 (36.84%)	1.00	1.00
Age of child (in months)	24-35	1 (0.09%)	294 (29.19%)	1.00	1.00
	36-47	6 (0.59%)	322 (31.98%)	5.48 (0.66, 45.77)	4.6 (0.53, 39.70)
	48-59	12 (1.19%)	372 (36.94%)	9.48 (1.23, 73.35)	9.81 (1.24, 77.36)^∗^
Sex of child	Female	11 (1.09%)	488 (48.46%)	1.41 (0.56, 3.53)	1.66 (0.18, 15.46)
	Male	8 (0.80%)	500 (49.65%)	1.00	1.00
Family size	≥4	17 (1.69%)	631 (62.66%)	4.81 (1.11, 20.93)	4.52 (1.02, 19.90)^∗^
	<4	2 (0.19%)	57 (35.45%)	1.00	1.00
Latrine availability	Yes	7 (0.69%)	210 (20.85%)	1.00	1.00
	No	12 (1.19%)	78 (77.26%)	2.16 (0.84, 5.55)	1.98 (0.75, 5.25)
Diarrhea in the last 2 weeks	Yes	6 (0.59%)	66 (6.55%)	6.45 (2.34, 17.22)	5.00 (1.73, 14.54)^∗^
	No	13 (1.29%)	922 (91.55%)	1.00	1.00
Respiratory infection in the last 2 weeks	Yes	(0.39%)	78 (7.74%)	3.11 (1.06, 10.17)	3.29 (1.07, 9.70)^∗^
	No	15 (1.49%)	910 (90.37%)	1.00	1.00

^∗^Statistically significant (*P* value < 0.05); unmarked = not significant.

**Table 7:** Results of bivariate and multivariable logistic regression of Bitot's spot among children aged 24-59 months in Lay Armachiho District, Central Gondar Zone, northwest Ethiopia, 2019.

Variables		Bitot's spot		COR (95% CI)	AOR (95% CI)
		Yes (%)	No (%)		
Age of child (in months)	24-35	3 (0.29%)	292 (28.99%)	1.00	1.00
	36-47	4 (0.39%)	324 (32.17%)	1.20 (0.27, 5.41)	1.21 (0.27, 5.47)
	48-59	15 (1.58%)	368 (36.54%)	3.96 (1.14, 13.79)	4.23 (1.13, 14.86)^∗^
Sex of the child	Male	15 (1.48%)	493 (48.95%)	2.14 (0.86, 5.27)	2.20 (0.89, 5.48)
	Female	7 (0.69%)	492 (48.86%)	1.00	1.00
Family size	≥4	18 (1.78%)	630 (62.56%)	2.54 (0.85, 7.55)	2.34 (0.77, 7.04)
	<4	4 (0.39%)	355 (35.25%)	1.00	1.00
Mother's educational status	Illiterate	17 (1.69%)	619 (61.47%)	2.01 (0.74, 5.49)	1.85 (0.67, 5.14)
	Literate	5 (0.49%)	366 (36.35%)	1.00	1.00
Handwashing practice after using the toilet	No	18 (1.79%)	570 (56.60%)	3.28 (1.10, 9.75)	3.02 (1.01, 9.13)^∗^
	Yes	4 (0.39%)	415 (41.21%)	1.00	1.00
Dietary	Adequate	6 (0.59%)	131 (13.00%)	1.00	1.00
Diversity	Inadequate	16 (1.59%)	854 (84.81%)	2.44 (0.94, 6.36)	2.18 (0.82, 5.81)

^∗^Statistically significant (*P* value < 0.05); unmarked = not significant.

**Table 8:** Prevalence of night blindness and Bitot's spot among children aged 24-59 months, Lay Armachiho District, Central Gondar zone, Northwest Ethiopia, 2019.

Variables		Frequency	Percent (%)
The difficulty of seeing during daytime	Yes	0	0
	No	1007	100
The difficulty of seeing during nighttime	Yes	19	1.9
	No	988	98.1
Both night blindness and Bitot's spot		8	0.79

## Data Availability

The datasets used and/or analyzed during the current study are available from the corresponding author on reasonable request.

## Abbreviations

ARI: Acute respiratory infection
DDS: Dietary diversity score
IMNCI: Integrated management of newborn and child illness
PCA: Principal component analysis
SPSS: Statistical Package for Social Sciences
VAD: Vitamin A deficiency
VADD: Vitamin A deficiency disorder
WHO: World Health Organization
X1B: Bitot's spot
XN: Night blindness.
